# Geniposide alleviates non‐alcohol fatty liver disease via regulating Nrf2/AMPK/mTOR signalling pathways

**DOI:** 10.1111/jcmm.15139

**Published:** 2020-04-15

**Authors:** Bingyu Shen, Haihua Feng, Jiaqi Cheng, Zheng Li, Meiyu Jin, Lilei Zhao, Qi Wang, Haiyan Qin, Guowen Liu

**Affiliations:** ^1^ Key Laboratory of Zoonosis Ministry of Education College of Veterinary Medicine Jilin University Changchun China

**Keywords:** adenosine 5’‐monophosphate‐ activated protein kinase, geniposide, non‐alcohol fatty liver disease, nuclear factor erythroid‐2‐related factor 2, oxidative stress

## Abstract

Non‐alcohol fatty liver disease (NAFLD) is a common disease which causes serious liver damage. Geniposide (GEN), a kind of iridoid glycoside extracted from *Gardenia jasminoides fruit*, has many biological effects, such as resistance to cell damage and anti‐neurodegenerative disorder. Lipid accumulation was obvious in tyloxapol‐induced liver and oil acid (OA) with palmitic acid (PA)‐induced HepG2 cells compared with the control groups while GEN improved the increasing conditions. GEN significantly lessened the total cholesterol (TC), the triglyceride (TG), low‐density lipoprotein (LDL), very low‐density lipoprotein (VLDL), myeloperoxidase (MPO), reactive oxygen species (ROS) and increased high‐density lipoprotein (HDL), superoxide dismutase (SOD) to response the oxidative stress via activating nuclear factor erythroid‐2–related factor 2 (Nrf2), haeme oxygenase (HO)‐1 and peroxisome proliferator‐activated receptor (PPAR)α which may influence the phosphorylation of adenosine 5’‐monophosphate–activated protein kinase (AMPK) signalling pathway in mice and cells. Additionally, GEN evidently decreased the contents of sterol regulatory element‐binding proteins (SREBP)‐1c, phosphorylation (P)‐mechanistic target of rapamycin complex (mTORC), P‐S6K, P‐S6 and high mobility group protein (HMGB) 1 via inhibiting the expression of phosphoinositide 3‐kinase (PI3K), and these were totally abrogated in Nrf2^−/−^ mice. Our study firstly proved the protective effect of GEN on lipid accumulation via enhancing the ability of antioxidative stress and anti‐inflammation which were mostly depend on up‐regulating the protein expression of Nrf2/HO‐1 and AMPK signalling pathways, thereby suppressed the phosphorylation of mTORC and its related protein.

## 1 INTRODUCTION

Non‐alcoholic fatty liver (NAFLD) is a disease that develops from hepatocyte steatosis or non‐alcoholic steatohepatitis (NASH) to liver fibrosis even liver cancer.^1^ During the process of NAFLD, it often occurs excessive liver and serum lipid accumulation with syndrome such as insulin resistance (IR), atherosclerosis and systemic inflammatory.^2^ In liver adipocytes, IR increases the rate of lipolysis leading to the ectopic of non‐esterified fatty acids (NEFA). Meanwhile, compensatory hyperinsulinaemia, which maintains normal glucose homeostasis, up‐regulates the transcription factor sterol regulatory element‐binding protein‐1c (SREBP‐1c) to drive the de novo lipogenesis.^3^ NAFLD occurs through NEFA catabolism to produce intrahepatic triglycerides, which are caused by mitochondrial oxidation and are exported as very low‐density lipoprotein (VLDL).^4^ The increasing NEFA is related to lipid toxicity, activation of inflammatory pathways and cytokine release.^5^ In the past few years, some new strategies have been proposed, such as regulating gene expression^6^ and key proteins associated with oxidative stress and inflammatory responses.^7^ One of the most important proteins is nuclear factor erythroid‐2–related factor 2 (Nrf2) from red blood cells,^8^ a nuclear factor, which was constitutively expressed in cytoplasm and inhibited by its negative regulator kelch‐like ECH (enoyl‐coa hydratase) related protein 1 (Keap1).^9^ Once the cells accept stimulation which could cause oxidative stress, the Keap1‐Nrf2 complex was disassembled and Nrf2 transferred into the nucleus to control the production of related enzymes such as haeme oxygenase 1 (HO‐1) and superoxide dismutase (SOD).^10^ In the process of Nrf2 working, phosphoinositide 3‐kinase (PI3K) controls the translocation^11^ and glycogen synthase kinase 3β (GSK3β) degrading the activity of Nrf2.^12^, ^13^ During the process of NAFLD, it often occurs a decreasing Nrf2 and applying natural antioxidants could prevent the oxidative stress by activating Nrf2 and related genes.^14^

PI3K‐AKT‐mTOR signalling pathway is a canonical signalling pathway in cell, which participates in regulating many critical functions, such as autophagy, cell proliferation and oxidative stress.^15^ Mechanistic target of rapamycin complex 1 (mTORC1) is an important growth regulator which is activated by nutrient cues, growth factors and cellular energy.^16^ It could be activated through class I PI3K‐mediated synthesis of phosphatidylinositol 3,4,5‐trisphosphate [PI (3,4,5) P3]. PI (3,4,5) P3 stimulates AKT, which contributes to activating of mTORC1. MTORC1 stimulates the synthesis of fatty acids and sterols by regulating the expression of SREBP‐1c, which is the master lipogenic transcription factor.^17^, ^18^, ^19^ mTORC1 could phosphorylate S6K and S6 and participate in regulation of autophagy. mTORC2 could be activated by growth factors and phosphorylate AKT (p‐ser473) in AGC kinase family to control various cellular processes.^20^

Geniposide (GEN) is a natural product extracted from the dry and ripe fruit of *gardenia*, which has many biological effects, such as anti‐inflammation and liver protection.^21^, ^22^ It has been reported that after AMPK knockout, GEN lost its protective effect against the obesity related cardiac injury.^23^ However, the specific mechanism of GEN on antioxidative stress and lipid accumulation induced by NAFLD in vivo or in vitro is not clear at present. The purpose of the research was to investigate the mechanism of GEN playing a role in NAFLD‐induced oxidative stress and inflammation, therefore to provide a theoretical basis for the development of new potential therapy on NAFLD.

## 2 MATERIALS AND METHODS

### 2.1 Chemical and regents

Geniposide (purity > 98%), penicillin and streptomycin, and FG ECL luminescence solution were provided by Dalian Meilun Biotechnology Co., Ltd. HepG2 cells were purchased from the China Cell Line Bank. Wild‐type (WT) C57BL/6 mice were provided by Liaoning Changsheng Technology Industrial Co., LTD. Nrf2^−/−^ (knockout) C57BL/6 mice were acquired from The Jackson Laboratory. Nrf2 (Cat.16396) and β‐actin (Cat.60008) antibodies were purchased from Proteintech. PPARα (ab8934), PPARγ (ab191407), P‐mTORC (phospho S2481, ab137133) and mTORC (ab2732) antibodies were provided by Abcam. Antibodies against HO‐1 (5853), AMPKα (5831), P‐AMPKα (Thr 172, 2535), AMPKβ (4150), P‐AMPKβ (Ser 182, 4186), adenosine acetyl‐CoA carboxylase (ACC) (3676), P‐ACC (Ser79, 11818), PI3K (4249), P‐p70 S6K (Thr389, 9234), p70 S6K (2708), P‐S6 (Ser235, 4858), P‐GSK 3β (9323) and GSK 3β (12456) were provided by Cell Signal Technology. AKT (AF6261), P‐AKT (Thr 308) (AF3262) and GAPDH (AF7021) were purchased from Affinity (OH, USA). S6 (abs131865) was provided by Absin. SREBP‐1c (NB600‐582) was provided by NOVUS (CO, US). HRP‐conjugated secondary antibodies (goat anti‐rabbit and goat antimouse) was obtained from Boster. Dulbecco's modified Eagle's medium (DMEM) was purchased from Invitrogen‐Gibco. Superior grade foetal bovine serum (FBS) was purchased from CLARK Bioscience. Mouse tumour necrosis factor (TNF)‐α, interleukin (IL)‐1β and IL‐6 ELISA kits were provided by Beijing Solarbio Science & Technology Co., Ltd. Total cholesterol (TC), triglyceride (TG), low‐density lipoprotein (LDL), high‐density lipoprotein (HDL), very low‐density lipoprotein (VLDL), myeloperoxidase (MPO), superoxide dismutase (SOD), reactive oxygen species (ROS), intercellular adhesion molecules 1 (ICAM‐1) and vascular cell adhesion molecule 1 (VCAM‐1), matrix metalloprotein (MMP)‐9 and monocyte chemoattractant protein 1 (MCP)‐1 ELISA kits were purchased from Nanjing Jiancheng Bioengineering Institute. Apolipoprotein C3 (ApoC3) ELISA kit was provided by Meimian Bio Institute.

### 2.2 Cell culture and cytotoxicity

HepG2 cells were cultured in DMEM containing 10% FBS, 100 U/mL penicillin and streptomycin at 37°C with 5% CO_2_. Cells were cultured in 96‐well plate and dealt with different concentrations of geniposide (0, 65, 130, 260, 390 and 520 μmol/L) for 24 hours with or without OA (660 μmol/L) and PA (330 μmol/L). Cells were subjected to cell counting kit (CCK)‐8 at 10 μL/well for 2 hours at 37°C. The optical density (OD) value 450 nm was read by a Multiskan FC (Thermo Scientific).

### 2.3 Cell oil red O staining

The cells were inoculated into 24‐well plate with sterile cell slides and treated with 260 μmol/L GEN with or without OA (660 μmol/L) and PA (330 μmol/L) for 18 hours. The slides were incubated with the ORO fixative for 25 minutes. Then, slides were washed by distilled water twice and soaked with 60% isopropanol for 5 minutes. ORO staining solution was added into the plates for 15 minutes. The slices were washed with distilled water till there was no ORO dye. Haematoxylin stain was added onto the slides for 1 minute next to washing the slides with distilled water according to the manufacturer's introduction (Solarbio). The slices were observed under a microscopy (Olympus).

### 2.4 Extracted the nucleus and cytoplasm proteins

HepG2 cells were placed onto the 6‐well plates. After adhered, the cells were treated with GEN (65, 130, 260 μmol/L) for 1 hour with OA (660 μmol/L) and PA (330 μmol/L) for 18 hours. The cells were scraped from the plates and the nucleus and cytoplasm protein were obtained by Nuclear and Cytoplasmic Protein Extraction Kit (Beyotime).

### 2.5 Western blotting

Total protein of cells was extracted using RIPA lysis buffer for about 5 minutes (Beyotime). The concentrations of proteins were detected by BCA kit (Thermo Pierce). About 20 μg extracted protein in every group was loaded onto 10% sodium dodecyl sulphonate polyacrylamide gel (SDS‐PAGE) and transferred onto polyvinylidene fluoride (PVDF) membrane. These membranes were incubated with primary antibodies (1:1000 or 1:500) overnight at 4°C and then incubated with HRP‐conjugated secondary antibodies (1:5000) for 1 hour at RT. Finally, the membranes were washed 4 times in TBST and observed through chemiluminescence Western blotting detection system. The grey scales of the bands were analysed using Image‐Pro Plus software.

### 2.6 Immunofluorescence

The cells were cultured onto 15 mm plate and dealt with 260 μmol/L GEN with OA and PA for 18 hours. The dishes were washed with PBS for 3 times and fixed by 4% paraformaldehyde (Solarbio) for 20 minutes. After washed with PBS for 15 minutes, proteinase K (1:1000 with PBS) was added into the dishes for 30 seconds prior to washed by PBS. 1 mL Triton X‐100 (1:1000 with PBS, Beyotime) was added into the cells for 10 minutes. Cells were incubated with the primary antibodies (1:200 with goat serum) overnight at 4°C. The hepatocytes were co‐incubated with secondary antibodies conjugated to Cy3 (Boster) in dark for 45 minutes. The nucleus was stained by DAPI (Solarbio) for 10 minutes. The slides were closed by coverslips and observed under a laser confocal microscopy (Fluoview FV1200, OLYMPUS).

### 2.7 Animals and ethics statement

The male wild‐type (WT) and Nrf2^−/−^ C57BL/6 mice (6‐8 weeks), weighing 18‐22 g, were housed in a specific pathogen free environment fed a normal diet (24 ± 1°C, relative humidity: 40%‐80%, n = 3 per cage). All experiments were carried out in an accordance with the National Institutes of Health (NIH) Guide for the Care and Use of Laboratory Animals and approved by the Jilin University animal administration committee.

### 2.8 Animal groups and procedures

All mice were randomly divided into 7 groups: Control; Tyloxapol (Ty); Ty (500 mg/kg) + GEN (50 mg/kg); Ty + GEN (75 mg/kg); Ty + GEN (100 mg/kg); GEN (100 mg/kg); Ty + Fenofibrate (100 mg/kg) groups. The mice in Ty + GEN groups, GEN only group and Ty + Fenofibrate group were injected GEN or fenofibrate prior 1 hour to administration of tyloxapol. After 18 hours, the mice were killed painlessly and then blood and liver were collected.

### 2.9 Biochemical detection

The level of TC, TG, LDL, HDL, VLDL, MPO, SOD, ROS, ICAM‐1, VCAM‐1, MMP‐9, ApoC3, MCP‐1, TNF‐α, IL‐1β and IL‐6 in blood of WT and Nrf2^−/−^ mice were detected by ELISA kits following the introduction of the manufacturer.

### 2.10 Western blotting

About 20 mg liver tissue of each mice was collected and made into homogenate in a 1.5 mL tube by Electric Tissue Grander (Tiangen OSE‐Y30). 300 μL RIPA lysis buffer was added into the tubes for 20 minutes. The following steps were performed as described before.

### 2.11 Immunohistochemistry

The liver tissue was fixed in 4% paraformaldehyde and placed in the embedding box. The blocks were located in 70% alcohol for the night, 80% alcohol for 2 hours, 90% alcohol for 1 hours, 95% alcohol for 40 minutes, anhydrous ethanol I for 40 minutes and anhydrous ethanol II for 30 minutes, respectively. The organization after dehydration was put into xylene II for 10 minutes and xylene II for 10 minutes. Tissue was paraffined for 2 hours, sliced and baked. The slices were putted into xylene for 16 minutes, anhydrous ethanol I for 20 minutes, 95% alcohol for 10 minutes, 90% alcohol for 10 minutes, 80% alcohol for 10 minutes and ddH_2_O for 10 minutes. The slices were dipped in the citrate buffer (PH 6.0), boiled and cooled for 10 minutes to repair antigen. The sections were placed in 3% hydrogen peroxide solution, incubated for 10 minutes and washed in dH2O for 5 minutes each time. 100‐400 μL goat serum was added blocking at room temperature for 1 hour. The sections were drop into the primary antibodies (Nrf2 and PPARα, 1:150 with goat serum) at 4°C overnight. TBST was used to clean the sections for 3 times, 5 minutes each. The second antibody solution (1:1000) was added onto the sections incubating in a wet box at room temperature for 30 minutes. Washed by TBST as before. Diaminobenzidine (DAB) was used to dye the slices. The slices were counterstained by haematoxylin for 20 minutes, rinsed by tap water and sealed. The slices were observed under a microscopy (Olympus). The negative controls were included in every experiment (using goat serum instead of primary antibodies).

### 2.12 Oil red O staining

The fresh liver tissues were embedded in optimal cutting temperature compound (OCT, SAKURA) and stored at −20°C. The tissue blocks were sliced into about 5 μm by a freezing microtome (Leica). The slices were stained by oil red O for 15 minutes and by haematoxylin staining for 30 seconds. The slices were washed under the running water and sealed with a cover glass. The slices were observed under a microscopy (Olympus).

### 2.13 Haematoxylin and eosin staining

The liver tissues were obtained, located into 4% paraformaldehyde and then embedded in paraffin. The wax blocks were cut into a thickness of 5 μm and stained with haematoxylin and eosin (H & E). The slices were observed under a microscopy (Olympus).

### 2.14 Statistical analysis

All experiments were all repeated at least 3 times. All data were expressed by Mean ± SEM and analysed by SPSS 19.0 (IBM). Comparisons between two groups were conducted by ONE‐WAY ANOVA, and comparisons between multiple groups were made by LSD method. *P* < .05 means significantly between two groups.

## 3 RESULTS

### 3.1 Geniposide had no effect on cell viability and reduced lipid accumulation in HepG2 cells

**Figure 1:**
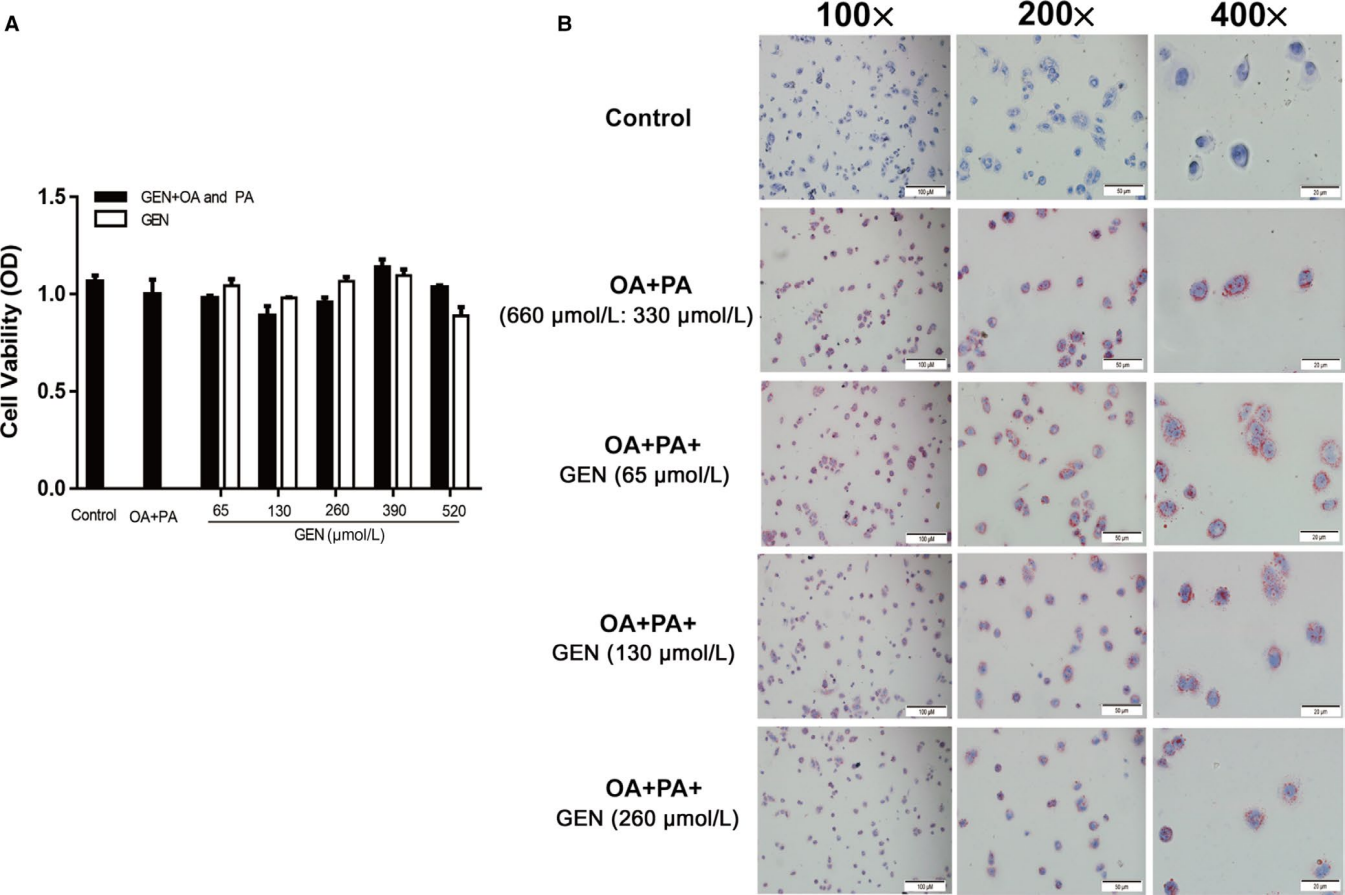
Effect of GEN on cytotoxicity and lipid accumulation in HepG2 cells. A, HepG2 cells were exposure to the different concentrations of GEN (0, 65, 130, 260, 390 and 520 μmol/L) with or without OA (660 μmol/L) and PA (330 μmol/L) for 24 h. The cell viability was tested by cell count kit (CCK)‐8. B, The effect of GEN (65, 130 and 260 μmol/L) on the lipid accumulation of HepG2 cells. The magnifications were 100×, 200× and 400×, and the scale bars, respectively, were 100, 50 and 20 μm

From Figure 1A, it could observe that when the concentrations of GEN were 0, 65, 130, 260, 390 and 520 μmol/L, there was no significance between the GEN group with or without OA + PA group and the control group which indicated that GEN (65‐520 μmol/L) had no toxicity for HepG2 cells. In Figure 1B, there was little lipid deposition in HepG2 cells. After OA (660 μmol/L) and PA (330 μmol/L) treatment, it appeared the excessive amounts of lipid in the cells. However, the lipid droplets in cells were significantly reduced after GEN application.

### 3.2 Geniposide activated Nrf2, PPARα and HO‐1 in HepG2 cells

**Figure 2:**
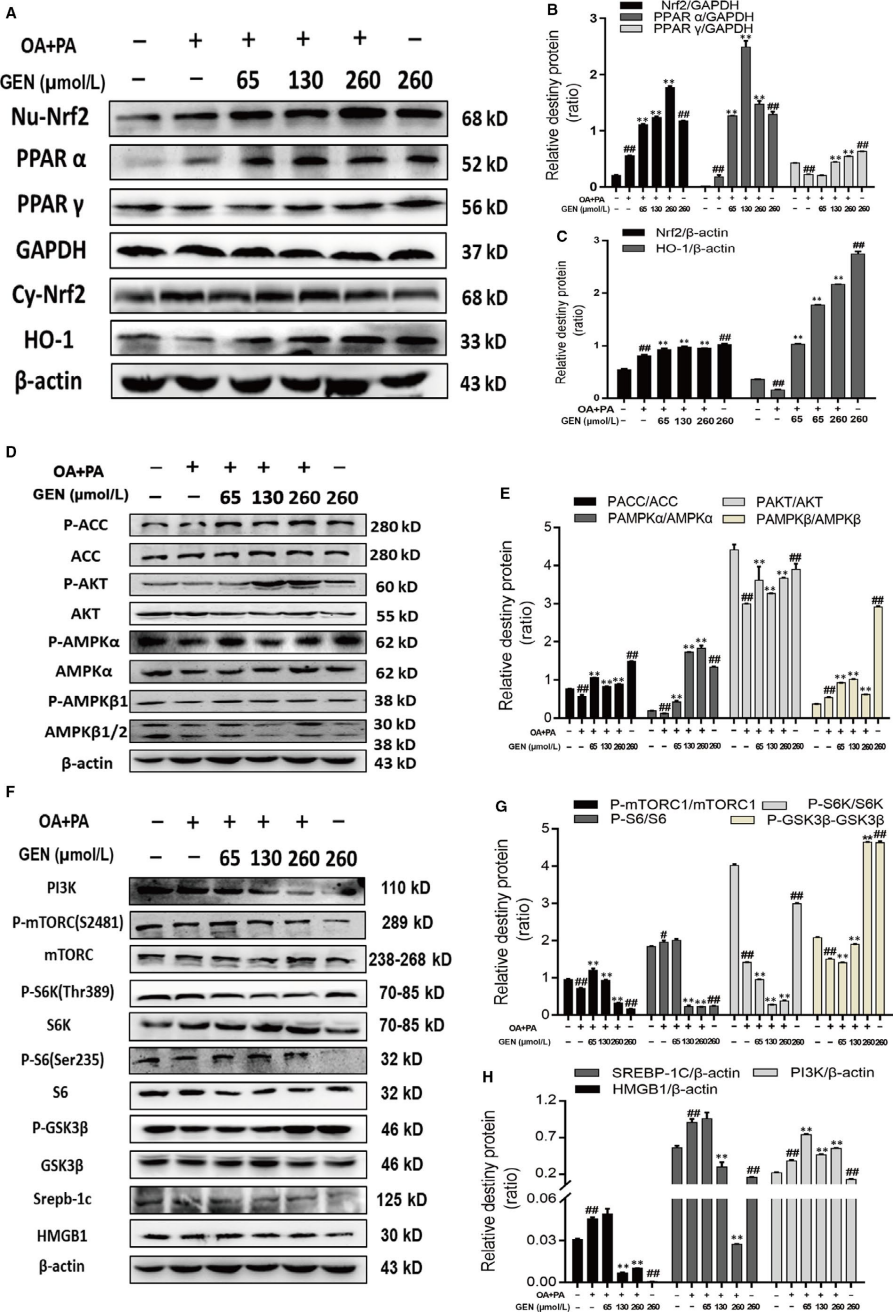
Effect of GEN on the expression of Nrf2‐related signalling proteins in OA (660 μmol/L) and PA (330 μmol/L)‐induced HepG2 cells. GEN was added into HepG2 cells prior 1 h to stimulation of OA and PA for 18 h. A‐C, Protein expression of Nrf2, PPARα, PPARγ in nucleus and HO‐1 in cytoplasm was detected by Western blot. (D and E) Protein expression of P‐ACC, ACC, P‐AKT, AKT, P‐AMPKα, AMPKα, P‐AMPKβ and AMPKβ was detected by Western blot. F‐H, Protein expression of PI3K, P‐mTORC, mTORC, P‐S6K, S6K, P‐S6, S6, SREBP‐1c and HMGB1 was detected by Western blot. The similar results were collected from three dependent experiments. All data were expressed by mean ± SEM (n = 5 in each group). ^##^ *P* < .01 vs Control Group; *P < .05 and **P < .01 vs OA and PA Group

**Figure 3:**
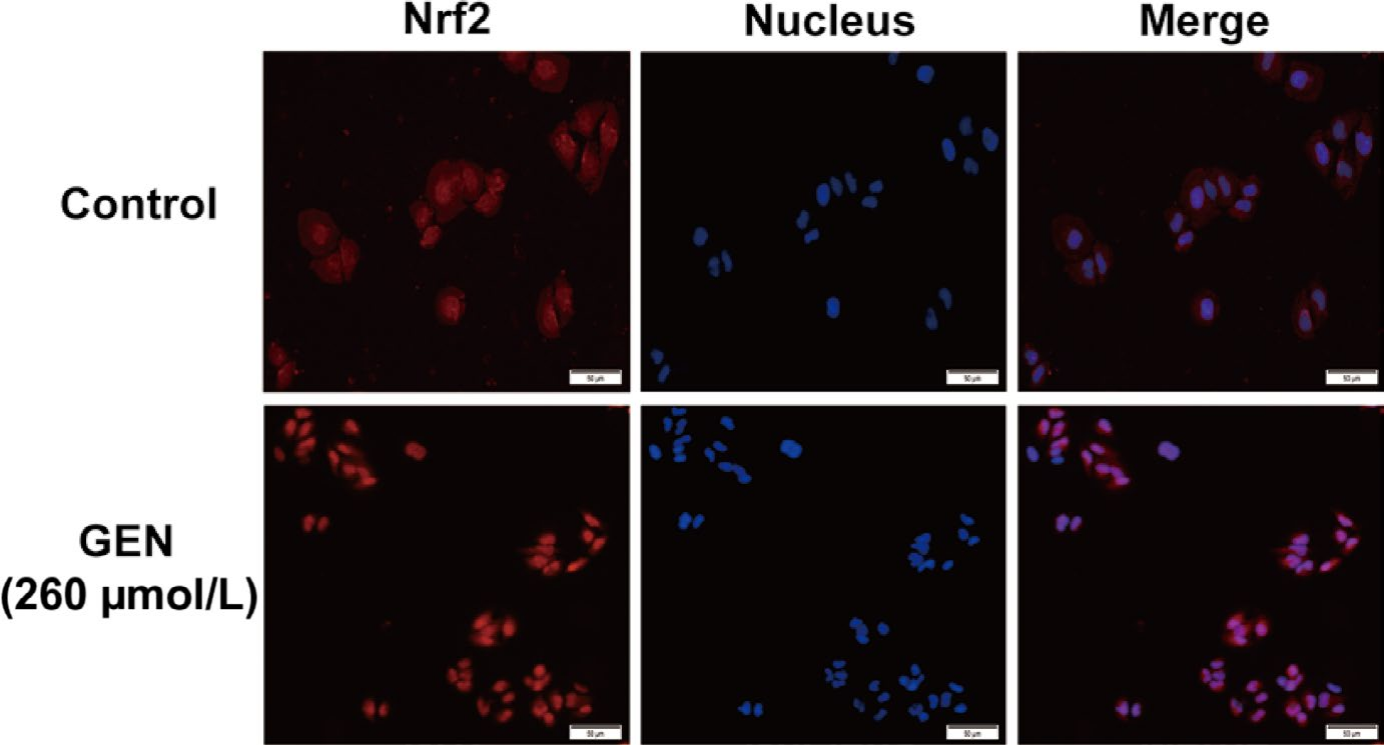
Effect of GEN on the Nrf2 transferring into the nucleus. GEN was added into HepG2 cells for 18 h and the transferring of Nrf2 (red) was detected by immunofluorescence. The nucleus was presented as blue, and the scale bars were 50 μm

Nrf2 plays a critical effect in antioxidative stress, and PPARα and PPARγ are also involved in regulating lipid metabolism. GEN (65, 130, 260 μmol/L) pre‐treatment facilitated the expression of Nrf2, PPARα and PPARγ in nucleus and then increased the protein content of HO‐1 in cytoplasm (Figure 2A‐C). In Figure 3, the results of immunofluorescence demonstrated that GEN assisted Nrf2 transferring into the nucleus in turn to control the downstream protein expression.

### 3.3 Geniposide regulated the AMPK/mTORC signalling pathways in HepG2 cells

As a key physiological energy sensor, AMPK is a major regulator of the energy balance of cells and organisms, co‐ordinating multiple metabolic pathways, balancing energy supply and demand, and ultimately regulating the growth of cells and organs. Regulation of energy metabolism balance is mediated by a number of related signalling pathways, among which AMPK/mTOR signalling pathway jointly constitutes a switch for anabolic and catabolic processes in cells. In addition, AMPK/mTOR signalling pathway is an important regulatory pathway of autophagy. In Figure 2D‐H, GEN elevated the phosphorylation of ACC, AKT, AMPKα, AMPKβ, GSK 3β and inhibited the levels of PI3K, P‐mTORC, P‐S6K, P‐S6, SREBP‐1c and HMGB1 in a dose‐dependent manner compared with the OA and PA group.

### 3.4 Geniposide alleviated the serum dyslipidaemia of mice

**Figure 4:**
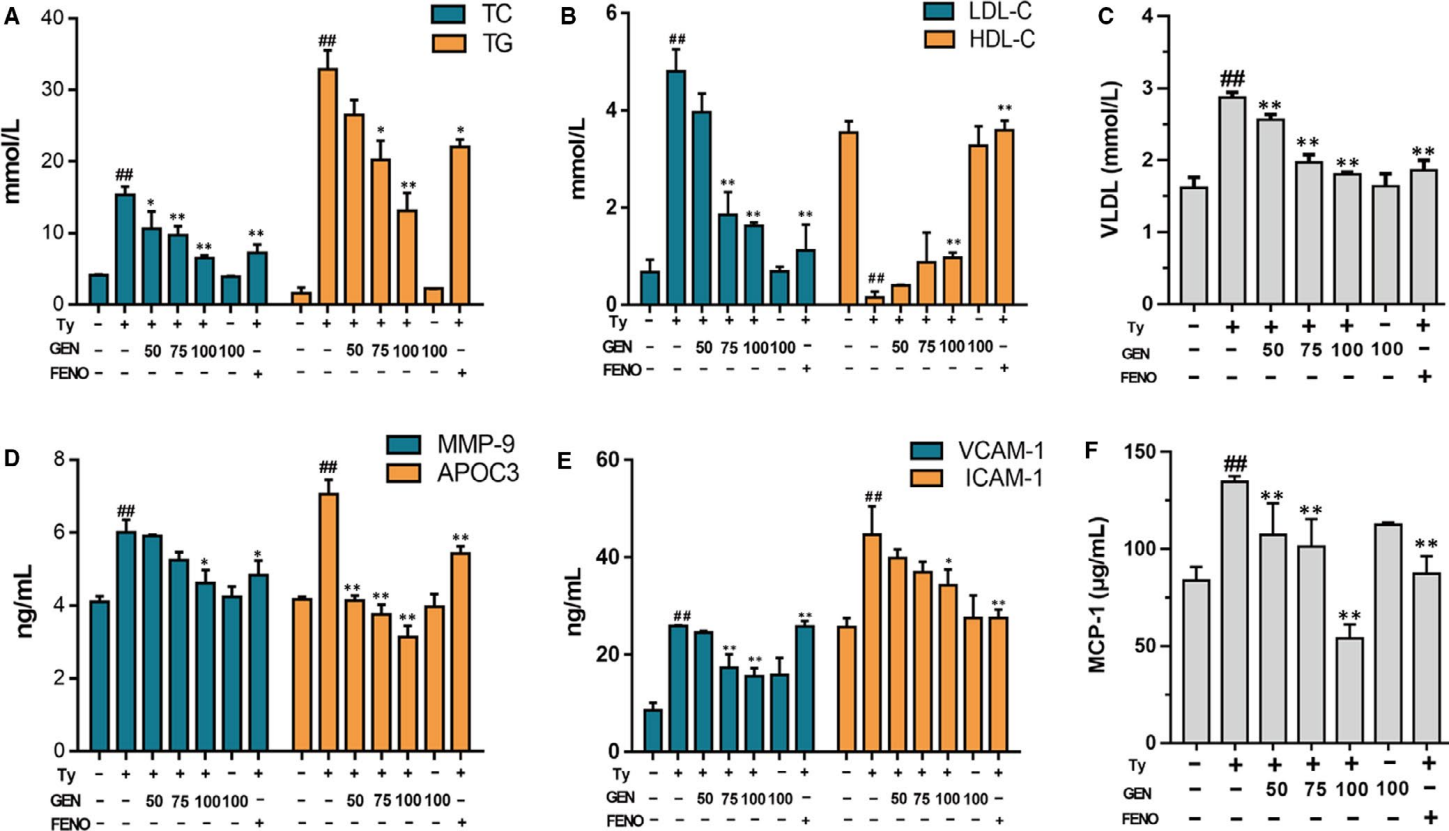
Effect of GEN on the release of TC, TG, LDL, HDL, VLDL, MMP‐9, ApoC3, ICAM‐1, VCAM‐1, MMP‐9 and MCP‐1 in tyloxapol‐induced mice. GEN (50, 75, 100 mg/kg) was administered to WT mice prior 1 h to tyloxapol (500 mg/kg)‐induced for 18 h. A, Effect of GEN on the content of TC and TG. B, Effect of GEN on the content of LDL and HDL. C, Effect of GEN on the content of VLDL. D, Effect of GEN on the content of MMP‐9 and ApoC3. E, Effect of GEN on the content of VCAM‐1 and ICAM‐1. F, Effect of GEN on the content of MCP‐1. The similar results were collected from three dependent experiments. All data were expressed by mean ± SEM (n = 5 in each group). ^#^ *P* < .05 and ^##^ *P* < .01 vs Control group; *P < .05 and **P < .01 vs Tyloxapol group

In Figure 4A,B,C, after tyloxapol induced, the contents of TC, TG, LDL and VLDL were increased significantly compared with the control group, indicated the occurrence of hyperlipidemia which may lead to NAFLD. However, GEN decreased the levels of TC, TG, LDL and VLDL and increased HDL in serum compared with the tyloxapol‐induced mice which suggested that GEN had the ability of reducing the serum lipid. Meanwhile, GEN also decreased the contents of MMP‐9, ApoC3, VCAM‐1, ICAM‐1 and MCP‐1 which participated in the adjusting the blood viscosity and the enrichment of inflammatory cells. While after Nrf2 knockout, the significant relief effect of GEN in TC, TG and LDL disappeared (Figure S1).

### 3.5 Geniposide up‐regulated the oxidative stress ability of mice and down‐regulated the inflammatory response induced by tyloxapol

**Figure 5:**
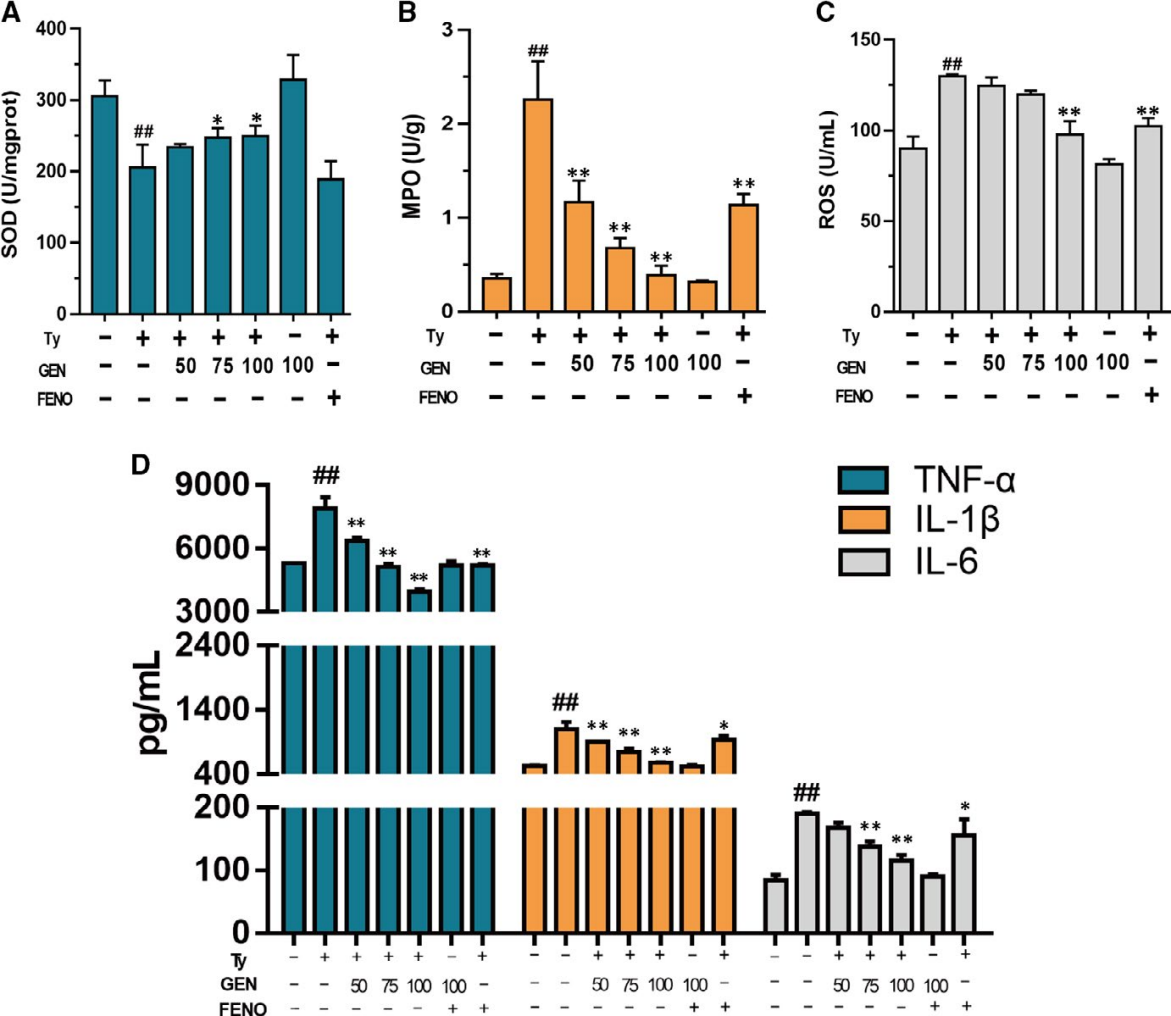
Effect of GEN on NAFLD oxidative stress and inflammation of tyloxapol‐induced mice. A, Effect of GEN on the content of SOD. B, Effect of GEN on the content of MPO. C, Effect of GEN on the content of ROS. D, Effect of GEN on the content of TNF‐α, IL‐1β and IL‐6. The similar results were collected from three dependent experiments. All data were expressed by mean ± SEM (n = 5 in each group). ^#^ *P* < .05 and ^##^ *P* < .01 vs Control group; *P < .05 and **P < .01 vs Tyloxapol group

As illustrated in Figure 5A‐C, compared with the tyloxapol group, GEN pre‐treatment elevated the content of SOD and reduced MPO and ROS, which suggested that GEN prevented the oxidative stress damage induced by tyloxapol via regulating the production of essential enzymes. GEN also decreased the over‐production of pro‐inflammatory cytokines TNF‐α, IL‐1β and IL‐6, proved GEN could reduce the inflammation during the progress of NAFLD.

### 3.6 Geniposide accelerated the expression of Nrf2, PPARα, PPARγ, HO‐1 and regulated the AMPK/mTORC signalling pathways in mice

**Figure 6:**
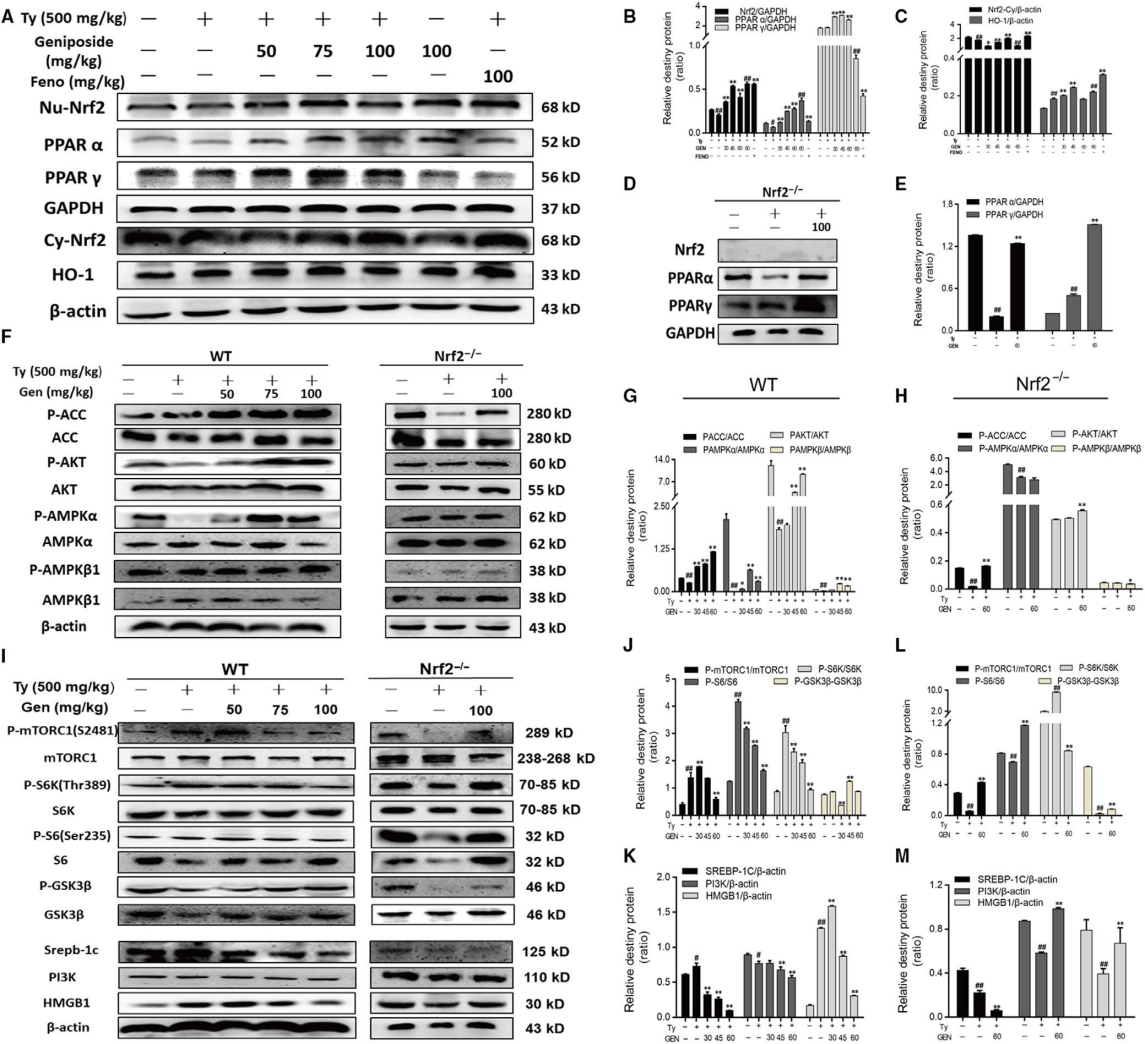
Effect of GEN on the protein expression of Nrf2, ACC and mTORC signalling pathways in tyloxapol‐induced mice. GEN (50, 75, 100 mg/kg) was administered to WT or Nrf2^−/−^ mice prior 1 h to stimulation of tyloxapol (500 mg/kg) for 18 h. A‐C, Protein expression of Nrf2, PPARα, PPARγ in nucleus and HO‐1 in cytoplasm of WT mice was detected by Western blot. (D and E) Protein expression of Nrf2, PPARα and PPARγ in Nrf2^−/−^ mice was detected by Western blot. F‐H, Protein expression of P‐ACC, ACC, P‐AKT, AKT, P‐AMPKα, AMPKα, P‐AMPKβ, AMPKβ in WT and Nrf2^−/−^ mice was detected by Western blot. I‐M, Protein expression of PI3K, P‐mTORC, mTORC, P‐S6K, S6K, P‐S6, S6, SREBP‐1c and HMGB1 WT and Nrf2^−/−^ mice was detected by Western blot. The similar results were collected from three dependent experiments. All data were expressed by mean ± SEM (n = 5 in each group). ^##^ *P* < .01 vs Control group; *P < .05 and **P < .01 vs Tyloxapol group

**Figure 7:**
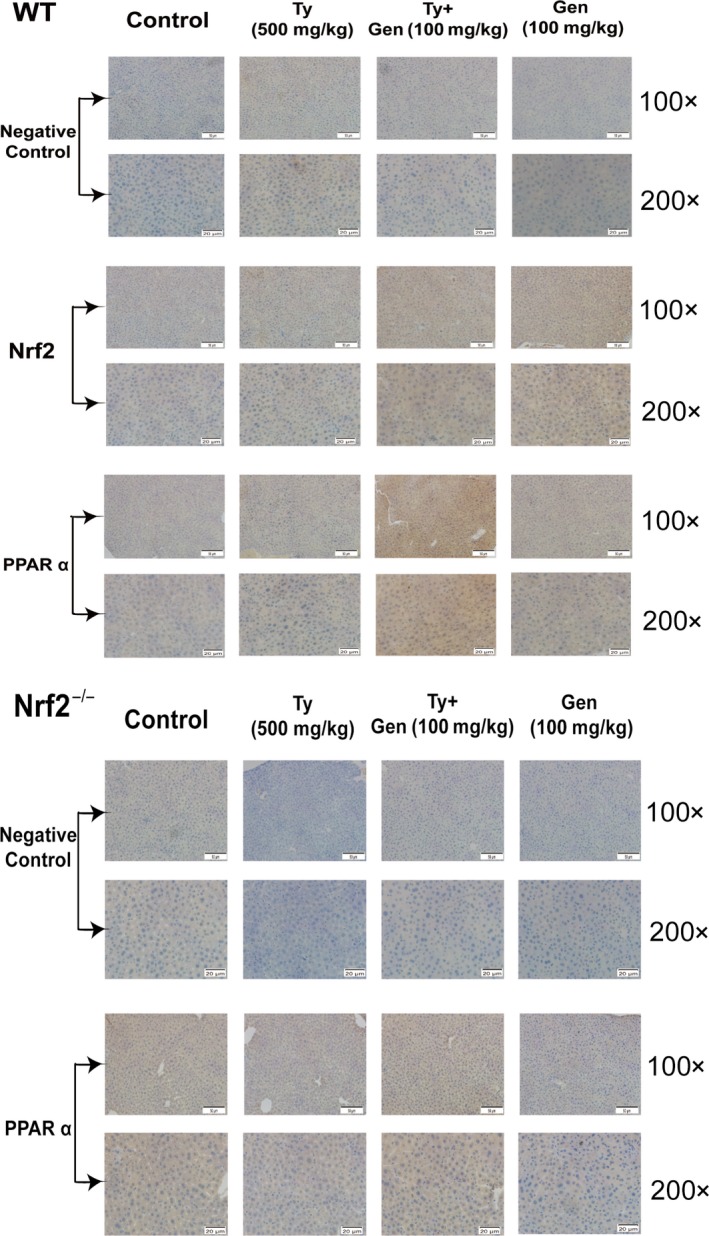
Effect of GEN on the expression of Nrf2 and PPARα in liver of WT and Nrf2^−/−^ mice. GEN (50, 75, 100 mg/kg) was administered to WT or Nrf2^−/−^ mice prior 1 h to stimulation of tyloxapol (500 mg/kg) for 18 h, and the expression of Nrf2 and PPARα in liver tissue was detected by immunohistochemistry (magnification ×100 and ×200)

Same as the results in vitro, GEN (50, 75, 100 mg/kg) could promote the proteins Nrf2 and PPARα into nucleus (Figure 6A‐C). The results of immunochemistry also verified the increasing expression (Figure 7). GEN also can facilitate the expression of HO‐1 in cytoplasm which suggested that GEN could protect liver from antioxidant damage. In addition, GEN promoted the phosphorylation of ACC, AKT, AMPKα, AMPKβ and inhibited the levels of P‐mTORC, P‐S6K, P‐S6 and SREBP‐1c in a dose‐dependent manner compared with the tyloxapol group. It probably happened through inhibiting the expression of PI3K and promoting the phosphorylation of GSK 3β whose expressions are influenced by Nrf2. Therefore, we applied GEN in the Nrf2 knockout mice stimulated by tyloxapol. It was found that the acceleration of P‐AKT, P‐GSK 3β and inhibitory effect on P‐mTORC, P‐S6, PI3K and HMGB1 after GEN treatment were weakened even disappeared (Figure 6D‐M). It further proved that GEN blocked NAFLD through activating Nrf2 and related protein expressions to answer oxidative stress.

### 3.7 Geniposide reduced the lipid accumulation and inflammation damages in tyloxapol‐induced mice

**Figure 8:**
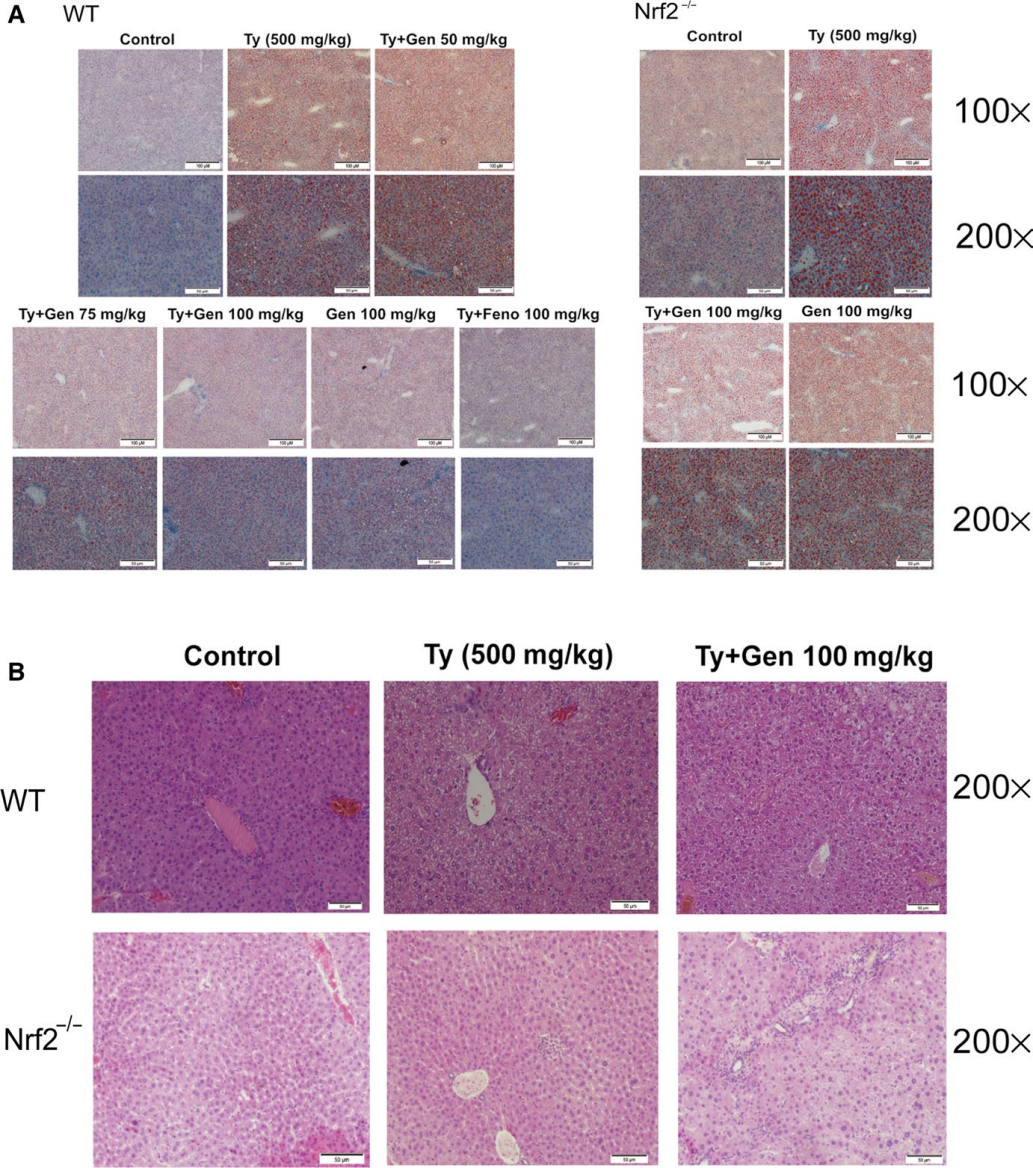
Effect of GEN on liver lipid accumulation and pathological changes of WT and Nrf2^−/−^ mice. GEN (50, 75, 100 mg/kg) was administered to WT or Nrf2^−/−^ mice prior 1 h to stimulation of tyloxapol (500 mg/kg) for 18 h. A, The lipid accumulation was detected by oil red staining (magnification 100× and 200×, red: lipid; blue: nucleus). B, Haematoxylin and eosin staining method was used to observe pathological changes. (magnification ×200)

As in Figure 8A, after tyloxapol induced, there was an amount of lipid accumulated in the hepatocyte in wild‐type and Nrf2^−/−^ mice, while GEN treatment (75, 100 mg/kg) ameliorated the elevation gradually. Haematoxylin and eosin staining was used to investigate whether inflammation histologic changes occur during NAFLD. In Figure 8B, tyloxapol caused the inflammatory cells infiltration and liver tissue loose compared with the control group, whereas pre‐treatment with GEN obviously attenuated these changes. In Nrf2^−/−^ mice, the pathologic changes were more obvious than in WT mice and the improvement ability of GEN was also been impaired after Nrf2 knockout.

What's more, in Nrf2^−/−^ mice, after tyloxapol induced, the levels of TC, TG and LDL were increased significantly compared with the tyloxapol group of WT mice while after application of GEN, the levels were not significant reduction. It indicated that the regulating serum lipid effect of GEN may play via Nrf2 (Figure S1).

## 4 DISCUSSION

NAFLD is the most common liver disease which acquired by metabolic stress liver injury, infected almost one‐third population in some areas.^24^ It is often seen as a manifestation of metabolic syndrome in the liver. The characteristic of NAFLD is excessive lipid accumulation in the liver which is not caused by alcohol. Patients with NAFLD increased the risk of type 2 diabetes and cardiovascular disease (CVD). At present, there is no specific treatment for NAFLD, other than weight and diet control. Therefore, it is critical to develop new drugs targeting NAFLD. In the research, geniposide was administrated to tyloxapol‐induced NAFLD C57BL/6C mice and FFA‐induced HepG2 cells to observe the specific mechanism of GEN on lipid accumulation. After tyloxapol or OA and PA induced, there was amount of lipid droplets in the liver cells dyed orange with oil red while GEN reduced intracellular fat production both in mice and cells (Figures 1B and 8A). The levels of TC, TG, LDL and VLDL were significantly increased in serum of tyloxapol‐induced mice compared with the control group and GEN pre‐treatment suppressed the increasing trends which indicated that tyloxapol could inhibit the action of lipolytic enzymes and the mechanism of lipoproteins. Following the lipid accumulation in the blood and liver, the adhesion intercellular and vascular was escalated which contribute to the development of NAFLD. However, GEN treatment reduced the lipoproteins in serum and alleviates the adhesivity of cells and vessel by bringing down VCAM‐1, ICAM‐1, MCP‐1 and rising up the level of HDL. In addition, animals knockout the gene of ApoC3 showed the lower TG and overexpression of ApoC3 tend to rise up the level of TG.^25^ In tyloxapol group, the level of ApoC3 significantly higher than that in control group while GEN pre‐treatment exhibited inhibiting ApoC3.

The liver has high metabolic activity and is the main source of liver metabolism detoxification of exogenous substances. These all increase the risk of placing the liver exposure to ROS and electrophilic. Transcription factor Nrf2 is a group of positive regulatory pressure factors involved in antioxidant/electrophilic protection of gene expression. Under normal physiological conditions, Nrf2 is anchored in the cytoplasm by Keap1. Keap1, as the substrate of the E3 ubiquitin ligase complex dependent on Cullin 3 (Cul3), promotes ubiquitination of Nrf2 and rapid degradation by proteasome. After the hepatocytes attacked by ROS or electrophilic, Nrf2 dissociates from Keap1 and combination with antioxidant response element (ARE) in the nucleus then activates the expression of the antioxidant enzyme gene regulated by Nrf2 such as HO‐1.^8^, ^26^ In Figures 2A and 6A, the content of Nrf2 decreased after the application of tyloxapol or OA with PA whereas the pre‐treatment of GEN significantly increased the level of Nrf2 which suggested that GEN could enhance the antioxidative stress ability of hepatocyte via improving Nrf2 transferring into the nucleus both in mice and cells. Meanwhile, the expression of HO‐1 in cytoplasm was increased observably. PPARs, whose functions including participating in antioxidative processes and inflammation, are also the up‐regulation of antioxidant enzymes such as SOD.^27^ In the mice injection with GEN, the protein content of PPARα and PPARγ were higher than that in tyloxapol group. The results of immunofluorescence verified GEN facilitated Nrf2 separated from Keap1 and entered into the cell nucleus (Figure 3).

It is critical for cells to eliminating cell injury which relies on the co‐ordination of multiple signalling pathways such as mTOR^15^ and AMPK pathway. AMPK is the key molecule of energy metabolism regulation, which contributes to energy of cell homeostasis. Its activity is often reflected by Thr172 phosphorylation, as well as the downstream target phosphorylation state, such as ACC.^28^ From Figures 2 and 6, we concluded that GEN enhanced the phosphorylation of AMPKα, AMPKβ, ACC and AKT compared with the tyloxapol treated group in WT mice. Meanwhile, with the activation of AMPKα and AMPKβ, the expression of downstream protein, P‐mTORC, P‐S6, P‐S6K and SREBP‐1c was impaired via inhibiting PI3K. The activation of AKT would promote the phosphorylation of GSK3β which plays an important role in regulating the energy and lipid metabolism.^29^ Therefore, the GEN treatment also increased the activity of GSK3β.

According to the previous results, we assumed that GEN inhibited NAFLD via Nrf2 and related protein. In the experiment, we applied Nrf2^−/−^ mice to further confirm the role of Nrf2 in GEN regulating NAFLD. We found that after Nrf2 knockout, GEN marginally reduced the contents of TC, TG and LDL than in WT mice (Figure S1). Furthermore, in Figure 6, GEN also could increase the phosphorylation of ACC, AKT and AMPK β and inhibit the level of P‐S6K and SREBP1‐c. However, it failed to control the content of P‐AMPKα, PI3K, P‐mTORC and P‐GSK3β. Immunohistochemistry results also confirmed the previous assumption. In WT mice, it was easily to observe an increased expression of Nrf2 and PPARα. Whereas the content of PPARα was down‐regulated after Nrf2 knockout which illustrated GEN restrained oxidative stress mostly through Nrf2 (Figure 7).

According to the ‘two‐hit’ hypothesis, during NAFLD, there is an increasing level of free fatty acid (FFA) in the adipocytes and lipid accumulation in hepatocyte resulting an inflammation damage in liver. During the NAFLD, the contents of pro‐inflammatory cytokines, such as TNF‐α, IL‐1β and IL‐6, were significantly higher than that in healthy mice (Figure 5D) confirmed the ‘second hit’ hypothesis.^30^ In addition, HMGB1 is a highly conserved protein which could induce inflammation once secreted outside the cell.^31^ In our study, tyloxapol raised the content of HMGB1 while GEN pre‐treatment improved the increasing HMGB1 which suggested that GEN may alleviate tyloxapol‐induced inflammation. Tyloxapol induced the liver inflammatory reaction which created inflammatory vacuoles in the liver and large number of inflammatory cells infiltrated in the interstitium. After GEN treatment, the liver structure recovered to the normal structure and reduced the inflammatory cells gathering. Furthermore, in liver of Nrf2^−/−^ mice, the structure was more porous than it in WT mice which suggested that Nrf2 knockout reduced the ability to resist oxidative stress of liver (Figure 8B).

## 5 CONCLUSIONS

As shown in Graphical Abstract, our study firstly demonstrated that geniposide was capable to protect mice and cells from NAFLD‐induced oxidative stress and inflammation which mostly depended on up‐regulating the Nrf2 and adjusting the protein expression of AMPK/PI3K/mTOR signalling pathways. This finding revealed the essential role of Nrf2 in geniposide inhibiting lipid accumulation and oxidative stress caused by NAFLD which would provide a potential therapeutic targeting at NAFLD and other liver metabolism diseases.

## CONFLICT OF INTEREST

The authors report no conflicts of interest. The authors alone are responsible for the content of this manuscript.

## AUTHORS’ CONTRIBUTIONS

BYS and GWL wrote the paper and performed the experiments; HHF and JQC performed the experiments; ZL and LLZ analysed the data; and MYJ, QW and HYQ contributed to design the experiments.

## Supporting information

Figure S1

## Data Availability

The data used to support the findings of this study are available from the corresponding author upon request.
