# Supplementary figures and images for: Geniposide alleviates non‐alcohol fatty liver disease via regulating Nrf2/AMPK/mTOR signalling pathways
Source: J Cell Mol Med. 2020 Apr 15;24(9):5097–108. doi: 10.1111/jcmm.15139 (PMC7205797; doi:10.1111/jcmm.15139)

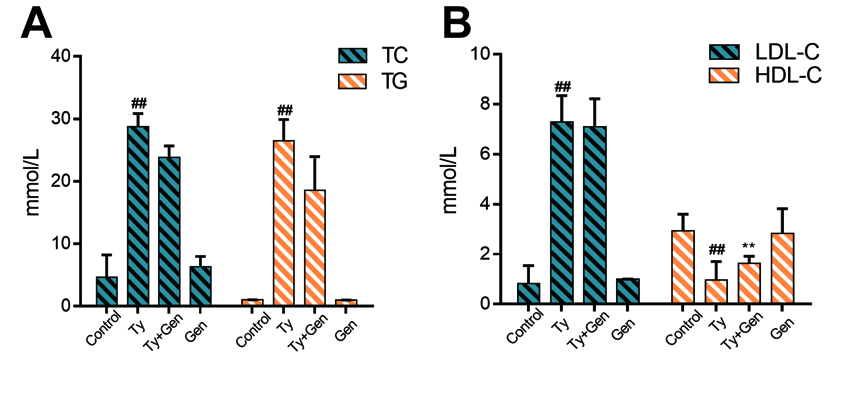

Supplement: Supplementary file 1 — Figure S1 [file JCMM-24-5097-s001.tif]
